# Photochemistry of *fac*-[Re(bpy)(CO)^3^Cl]**

**DOI:** 10.1002/chem.201202734

**Published:** 2012-10-18

**Authors:** Shunsuke Sato, Yasuo Matubara, Kazuhide Koike, Magnus Falkenström, Tetsuro Katayama, Yukihide Ishibashi, Hiroshi Miyasaka, Seiji Taniguchi, Haik Chosrowjan, Noboru Mataga, Naoto Fukazawa, Shinya Koshihara, Ken Onda, Osamu Ishitani, In memory of Professor Mataga

**Affiliations:** a Department of Chemistry, Graduate School of Science and Engineering, Department of Chemistry and Material Science, Graduate School of Science and Engineering, Tokyo Institute of TechnologyO-okayama 2-12-1, E1-9, Meguro-ku, Tokyo 152-8551 (Japan) E-mail: Ishitani@chem.titech.ac.jp; b National Institute of Advanced Industrial Science and TechnologyOnogawa 16-1, Tsukuba 305-8569 (Japan); c Institute for Laser TechnologyUtsubo-Honmachi 1-8-4, Nishi-ku, Osaka 550-0004 (Japan); d Division of Frontier Materials Science, Graduate School of Engineering Science, Center for Quantum Science and Technology under Extreme Conditions, Osaka UniversityToyonaka, Osaka 560-8531 (Japan) E-mail: miyasaka@chem.es.osaka-u.ac.jp; e CREST, Japan Science and Technology Agency (JST)Kawaguchi-shi, Saitama 332-0022 (Japan); f Interactive Research Center of Science, Tokyo Institute of TechnologyNagatsuta, Midori-ku, Yokohama, Kanagawa 226-8502 (Japan); g PRESTO, JST4-1-8 Honcho, Kawaguchi, Saitama 332-0012 (Japan); h ALCA, JST, SanbanchoChiyoda-ku, Tokyo 102-0075 (Japan)

**Keywords:** isomerization, ligand substitution, photochemistry, photophysics, rhenium

## Abstract

The photochemistry of *fac*-[Re(bpy)(CO)_3_Cl] (**1 a**; bpy=2,2′-bipyridine) initiated by irradiation using <330 nm light has been investigated. Isomerization proceeded in THF to give the corresponding *mer-*isomer **1 b**. However, in the presence of a small amount of MeCN, the main product was the CO-ligand-substituted complex (*OC*-6-24)-[Re(bpy)(CO)_2_Cl(MeCN)] (**2 c**; bpy=2,2′-bipyridine). In MeCN, two isomers, **2 c** and its (*OC*-6-34) form (**2 a**), were produced. Only **2 c** thermally isomerized to produce the (*OC*-6-44) form **2 b**. A detailed investigation led to the conclusion that both **1 b** and **2 c** are produced by a dissociative mechanism, whereas **2 a** forms by an associative mechanism. A comparison of the ultrafast transient UV-visible absorption, emission, and IR spectra of **1 a** acquired by excitation using higher-energy light (e.g., 270 nm) and lower-energy light (e.g., 400 nm) gave detailed information about the excited states, intermediates, and kinetics of the photochemical reactions and photophysical processes of **1 a**. Irradiation of **1 a** using the higher-energy light resulted in the generation of the higher singlet excited state with *τ*≤25 fs, from which intersystem crossing proceeded to give the higher triplet state (^3^HES(**1**)). In THF, ^3^HES(**1**) was competitively converted to both the triplet ligand field (^3^LF) and metal-to-ligand charge transfer (^3^mLCT) with lifetimes of 200 fs, in which the former is a reactive state that converts to [Re(bpy)(CO)_2_Cl(thf)]^+^ (**1 c**) within 10 ps by means of a dissociative mechanism. Re-coordination of CO to **1 c** gives both **1 a** and **1 b**. In MeCN, irradiation of **1 a** by using high-energy light gives the coordinatively unsaturated complex, which rapidly converted to **2 c**. A seven-coordinate complex is also produced within several hundred femtoseconds, which is converted to **2 a** within several hundred picoseconds.

## Introduction

The photochemistry and photophysics of the rhenium(I) complex *fac*-[Re(bpy)(CO)_3_Cl] (**1 a**; bpy=2,2′-bipyridine) have generated substantial interest because this complex emits from the triplet metal-to-ligand charge-transfer (MLCT) excited state even in solution at ambient temperature^1^ and can function as a photocatalyst for CO_2_ reduction.^2^ Recently, the application of **1 a** and its derivatives in electroluminescence processes^3^ and dye-sensitized solar cells^4^ has been reported, and it has also been used as a building block for various photoactive supramolecular systems.^5^ The photostability of **1 a** is one of the key reasons why this type of rhenium(I) diimine complex has been used so often in various research applications. In fact, the decomposition of **1 a** does not occur by irradiation when using 366 nm or longer wavelength light in either solution or crystal form.^6^ Therefore, only limited information on the excited states of **1 a** has been reported except for the lowest ^3^mLCT excited state from which energy- and electron-transfer reactions proceed.1f, h, 2d,^6^, ^7^ Recently, ultrafast emission spectroscopy was applied to **1 a**, and the kinetics of intersystem crossing from the singlet MLCT excited state and vibrational relaxation processes of the hot triplet states have been reported.8a, e

We recently reported both the photochemical ligand substitution9a and isomerization9b reactions of **1 a** for the first time. Irradiation of a solution of **1 a** in MeCN (<330 nm wavelength light) causes photochemical ligand substitution, thus giving two isomers: (*OC*-6-34)*-*[Re(bpy)(CO)_2_Cl(MeCN)] (**2 a**) and (*OC*-6-24)-[Re(bpy)(CO)_2_Cl(MeCN)] (**2 c**) [Eq. (1)]. One of the isomers (**2 a**) is both thermally and photochemically stable, whereas interconversion between the Cl^−^ and CO ligands of **2 c** proceeds thermally to give the third isomer, (*OC*-6-44)*-*[Re(bpy)(CO)_2_Cl(MeCN)] (**2 b**) [Eq. (2)]. On the other hand, in THF, the photochemical isomerization of **1 a** proceeds by irradiation (<330 nm wavelength light) to give the *mer* isomer (**1 b**) [Eq. (3)]. Since these products do not emit at room temperature, these reactions are potentially deactivation processes of **1 a** as a photocatalyst and as a photofunctional material.


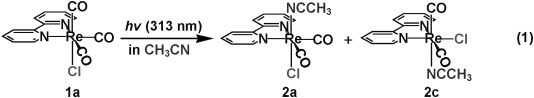
(1)


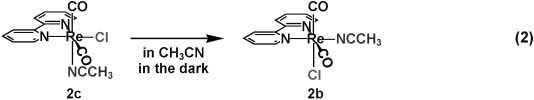
(2)


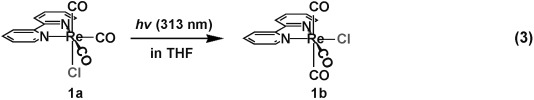
(3)

These photochemical reactions of **1 a** showed the following significant features: 1) the presence of O_2_ does not affect the rate of the disappearance of starting complex **1 a** during the photochemical reactions. 2) The quantum yields of the photochemical reactions are strongly dependent on the wavelength of light used for irradiation (i.e., light with energy higher than 330 nm, which corresponds to the π–π* absorption and/or the relatively higher-energy portion of the MLCT absorption, induces both photochemical reactions), whereas these reactions do not occur under irradiation with 365 nm light, which corresponds to the lower-energy part of the MLCT absorption. 3) Time-resolved IR measurements in MeCN indicate that the photochemical ligand substitution of **1 a** is complete within 50–100 ps. These facts clearly show that both the photochemical ligand substitution and isomerization reactions do not proceed through the lowest ^3^mLCT excited state. Therefore, a more detailed study of these photochemical reactions should initiate more thorough investigations of other higher excited states of **1 a**.

Here, we report a comprehensive evaluation of the photochemistry of **1 a**, in which the characteristics of these photochemical reactions are connected with kinetic and structural information on the short-lived excited states of **1 a** obtained by ultrafast time-resolved (TR) UV-visible and IR absorption, and emission spectroscopy.

## Experimental Section

**Materials**: THF was distilled from Na/benzophenone just before use. Acetonitrile was dried three times over P_2_O_5_ and was then distilled from CaH_2_ prior to use. Other reagents were purchased as reagent grade and were used without further purification. The complex *fac-*[Re(bpy)(CO)_3_Cl] **(1 a)** was synthesized according to previously reported methods.1d

**Photochemical reactions**: A solution of 1.0 mm **1 a** (4.0 μmol, 4 mL) was irradiated with 313 nm light using an Ushio 500 W high-pressure Hg lamp with an Asahi Spectra band-pass filter/313 nm. A gentle stream of argon was bubbled into the solution for about 15 min, and then the solution was photolyzed. The solution was kept at (25±1) °C during irradiation using a temperature control unit (TAITEC LbBath LB-21 JR). This unit was also used for temperature-dependence experiments. The disappearance of **1 a** and the formation of the products were followed by HPLC using a Shimadzu LC-10AD*VP* pump with a Develosil ODS-UG-5 column (250 mm×4.6 mm), a Rheodyne 7125 injector, a Shimadzu FCV-10AL*VP* gradient unit, and a Shimadzu SPD-10AV*VP* detector. The column temperature was kept at 297 K using a Shimadzu CTO-10AC*VP* oven, and a 1:1 (v/v) mixture of MeOH and a KH_2_PO_4_–NaOH buffer (0.05 m; pH, 5.9) was used as the eluent.

**DFT calculations**: Geometry optimization and frequency analyses in the gas phase were carried out for the electronic ground state of [Re(bpy)(CO)_2_Cl] at the B3LYP^10^, ^11^ level of theory by employing the Firefly QC package,12a which is partially based on the GAMESS (US)12b source code. The Hay–Wadt VDZ (*n*+1) effective core potentials and basis sets (LANL2DZ)^13^ were used for the Re atom. The Pople 6-31G* basis set with a pure d function^14^ was used for C, H, N, O, and Cl atoms. The local minima were confirmed by frequency analyses.

All solutions were bubbled with N_2_ or Ar gas before the following time-resolved spectroscopic experiments.

**Fluorescence upconversion measurement**: Time profiles of the fluorescence in the sub-picosecond to 100 ps time region were measured by using a homemade fluorescence up-conversion apparatus.^15^ A Ti:sapphire laser system (Verdi-V8 pumped Mira 900, Coherent, Inc.) was used as a light source (120 fs, 76 MHz, 800 mW at 820 nm). The pulses were further compressed up to approximately 70 fs fwhm using a prism pair compressor. The second harmonic (ca. 20 mW) was generated in a 0.1 mm-thin BBO crystal and was focused onto the sample circulating in a flow cell (50 mL min^−1^) with a 1 mm light path length to generate the fluorescence. This was collected by a pair of parabolic mirrors and focused, together with the residual fundamental laser pulse, on a 0.4 mm BBO type I crystal to generate the up-converted signal at the sum frequency. After passing through a grating monochromator, the signal was detected by a photomultiplier (R1527P) coupled with a photon counter (C5410) system (both from Hamamatsu Photonics). As an instrumental response function, the cross-correlation signal between the fundamental and its second harmonic pulses was used (fwhm≍130 fs).

**Time-resolved UV/Vis absorption spectroscopy**: A femtosecond dual NOPA/OPA laser system was used for transient absorption spectroscopy.^16^ The output of a femtosecond Ti:sapphire laser (Tsunami, Spectra-Physics) pumped by the second harmonic generation (SHG) of a continuous wave (cw) Nd^3+^:YVO_4_ laser (Millennia Pro, Spectra-Physics) was amplified with a 1 kHz repetition rate by using a regenerative amplifier (Spitfire, Spectra-Physics). The amplified pulse (802 nm, 0.9 mJ pulse^−1^, 85 fs fwhm, 1 kHz) was divided into two pulses with the same energy (50 % each). One of the two pulses was guided into a NOPA system (TOPAS-white, Light-Conversion), which covers the wavelength region between 500 and 780 nm with 1–40 mW output energy with approximately 20–40 fs fwhm. The wavelength of the NOPA was frequency-doubled by a 100 μm BBO crystal and used as the excitation laser pulses (UV region: 270 and 360–370 nm). After compression by a prism pair, the SHG in the UV region was used as a pump pulse with the intensity of 0.1 μJ pulse^−1^ at 270 nm and 0.2 μJ pulse^−1^ at 360–370 nm. The pulse duration at the sample position was estimated to be approximately 50 fs fwhm by FROG signals. The other pulse at 802 nm was guided into an OPA system (OPA-800, Spectra-Physics) and was converted to a 1200 nm pulse, which was focused on a 3 mm CaF_2_ plate to generate a white-light continuum covering the wavelength region from 350 to 1000 nm. This white light was used as a probe pulse. The polarization angle between the pump and the probe pulses was set at the magic angle for all measurements. The probe pulse was divided into signal and reference pulses, detected with multichannel photodiode array systems (PMA-10, Hamamatsu), and sent to a personal computer for further analysis. The chirping of the monitoring white light continuum was corrected for transient absorption spectra. The fwhm of the cross correlation between the pump and probe pulses was approximately 100 fs at the sample position. A rotational cell was used for femtosecond transient absorption and up-conversion measurements. The optical path length was 100 μm. Fused quartz plates with 1 mm thickness were used for windows of the rotational cell. Absorbance values at 370 and 270 nm were 0.80 and 0.82, respectively. The kinetic data were analyzed by linear or nonlinear least-squares methods by using commercial software (Igor Pro, Wave Metrics) and homemade programs. The details have been reported elsewhere.^16^

**Time-resolved IR spectroscopy**: Time-resolved IR (TR-IR) measurements were carried out by using a femtosecond broadband mid-IR pulse and a multi-channel IR detector equipped with a polychromator.^17^ The broadband mid-IR pulse was generated by optical parametric amplification (OPA) and difference frequency generation (DFG) from a part of the output of a Ti:sapphire regenerative amplifier (center wavelength: 800 nm; pulse duration: 120 fs; repetition rate: 1 kHz). The energy width and tunable range of the IR pulse were 150 cm^−1^ and 1000–3500 cm^−1^, respectively. The sample excitation pulse was generated by doubling (400 nm) or tripling (266 nm) the energy of the output from the other part of the amplifier. The typical energies were 8 and 4 μJ pulse^−1^ for 400 and 266 nm, respectively. The delay time between the excitation pulse and the IR probe pulse was controlled by an optical delay line. The transient IR absorption spectrum was obtained with a liquid-nitrogen-cooled 64-element mercury–cadmium–telluride (MCT) array using a polychromator with a focal length of 19 cm (FPAS, Infrared Systems Development Co.). To obtain a better signal-to-noise ratio, the excitation pulse train was chopped alternately with an optical chopper, and the difference between the IR spectra with and without pump pulses was collected. The sample solution flowed inside an IR cell with CaF_2_ windows. The path length of the IR cell was 0.5 mm and the absorbance at this path length at 400 and 266 nm were approximately 0.2 and 2.0, respectively. The spot diameters of the pump and probe pulses at the sample were 300 and 150 μm, respectively.

## Results and Discussion

**Figure 1:**
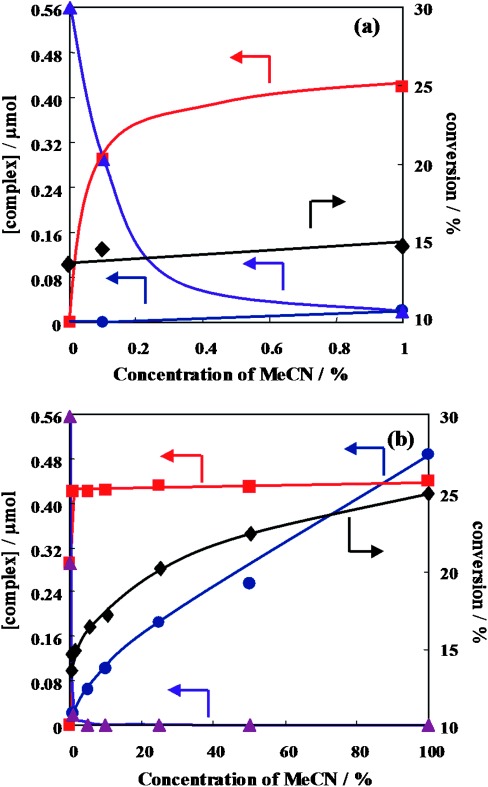
Dependence of the formation of **1 b** (▴), **2 a** (•), and **2 b** (▪) and the conversion of **1 a** (⧫) on the concentration of MeCN in the solutions of **1 a** (4.0 μmol) in THF–MeCN (4 mL). The solutions were irradiated using 313 nm light under an Ar atmosphere for 30 min: concentration of MeCN was a) 0–1 % and b) 0–100 %.

**Photochemistry in mixed solvents of THF and MeCN**: As we have already reported, irradiation of solution of **1 a** in THF caused the isomerization of **1 a** to the corresponding meridional isomer (**1 b**).9b However, the photochemical isomerization of **1 a** was drastically suppressed by the addition of a small amount of MeCN into the reaction solution. Figure 1 shows the dependence of the photochemical reaction of **1 a** (0.4 μmol) on the concentration of MeCN in mixed solutions of THF–MeCN under an Ar atmosphere for 30 min. Although the yield in the formation of **1 b** was 93 % in THF (based on the amount of **1 b** consumed), the addition of only 0.1 % MeCN to the solution caused a decrease in the yield of **1 b** to 49 % and the formation of one of the ligand-substitution products (**2 b**) in 49 % yield (Figure 1a). In a mixed solution of 99 % THF and 1 % MeCN, the yield for the formation of **1 b** was less than 1 % and that of **2 b** was 71 %. Note that in the mixed solution, the rate of the disappearance of **1 a** did not change relative to that in THF, and **2 a** was hardly observed in the mixed system up to a concentration of 1 % MeCN. In a mixed THF solution with 5 % MeCN, the main product was still **2 b**, whereas **2 a** formed in 9 % yield based on the consumption of **1 a**. Higher concentrations of MeCN caused an increase in both the consumption of **1 a** and the formation of **2 a** but did not affect the formation of **2 b** (Figure 1b). For example, in a 1:1 THF–MeCN mixed solution, irradiation for 30 min caused a 0.92 μmol decrease in **1 a** and the formation of **2 a** and **2 b** in 0.26 and 0.43 μmol yields, respectively, whereas 0.52 μmol of **1 a** was consumed in the THF solution that contained 1 % MeCN, thus giving 0.42 μmol of **2 b** with a very small amount of **2 a**, as described above.

The precursor of **1 b** should be the same as that of **2 b** because the increase in the formation of **2 b** (0.29 μmol) was similar to the decrease in the formation of **1 b** (0.27 μmol) by the addition of only 0.1 % MeCN into the THF solution (Figure 1a), and the addition of more than 1 % MeCN did not cause an increase in the photochemical formation of **2 b** (Figure 1b). These results also strongly suggest that the formation of both **1 b** and **2 b** proceeds by means of a dissociative mechanism. On the other hand, **2 a** should form through an associative mechanism because the yield of **2 a** was strongly dependent on the concentration of MeCN in the reaction solution.

The designated structures of the precursors and the suggested reaction mechanism are also strongly supported by the following experiment, in which a solution of **1 a** in acetonitrile was irradiated with 313 nm light under a CO atmosphere. In comparison with the results obtained under an Ar atmosphere, both the rate of the disappearance of **1 a** and that of the formation of **2 b** decreased by 15 %, but the rate of the formation of **2 a** did not change (see Figure S1 in the Supporting Information). Another important difference was that isomerization product **1 b** formed even in a neat MeCN solution under a CO atmosphere with 8 % yield, whereas **1 b** was not produced by irradiation in MeCN under Ar. These results clearly indicate that the precursor of **2 a** cannot react with CO, whereas that of **2 b** reacts with CO to give both **1 a** and **1 b**. Therefore, the photochemical-induced formation of **1 b** and **2 b** should proceed through the formation of the coordinatively unsaturated complex [Re(bpy)(CO)_2_Cl], and furthermore, a seven-coordinate intermediate is the potential precursor of **2 a**.

**Figure 2:**
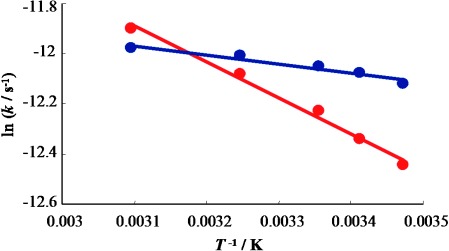
Arrhenius plots for the formation of **2 a** (blue) and **2 b** (red) by irradiation using 313 nm light in a solution of 1.0 mm **1 a** in MeCN under an Ar atmosphere. The yield of **2 b** was obtained after **2 c** was completely converted to **2 b** in the dark.

The temperature dependence of the rate of formation of **2 a** was also very different from that of **2 b** (Arrhenius plots are shown in Figure 2). The activation energies (*E*_a_) for the disappearance of **1 a** were (569±53) cm^−1^ in MeCN and (781±14) cm^−1^ in THF (see Figures S2 and S3 in the Supporting Information). In neat MeCN, the formation rate of **2 b** was also dependent on temperature (*E*_a_=(1112±58) cm^−1^), whereas a much smaller temperature dependence was observed for the formation of **2 a** in neat MeCN (*E*_a_=(131±110) cm^−1^). It should be noted that the *E*_a_ value for the formation of **2 b** in MeCN was similar to that for the disappearance of **1 a** obtained in THF.^18^ This also supports the idea that the formation of **1 b** and **2 b**, which is the thermal isomerization product of **2 c**, proceeds through the same intermediate (i.e., the coordinatively unsaturated complex).

It is worth noting that these activation energies are much smaller than those of the photochemical ligand substitution of *fac*-[Re(bpy)(CO)_3_(PR_3_)]^+^ (≍3500 cm^−1^), which proceeds by means of thermal activation from the lowest ^3^mLCT excited state to the triplet ligand field (^3^LF) excited state.^6^

**Mechanisms of the photochemical reactions**: All the results related to the photochemical ligand-substitution reactions described above (i.e., the dependence on the concentration of MeCN, the effect of the presence of CO, and the temperature dependence) clearly indicate that the mechanisms for the photochemical formation of **2 a** and **2 b** are different. On the other hand, the precursor of **1 b** produced by the photochemical isomerization of **1 a** in THF should be the same as that of **2 b**, as supported by the following results: 1) The addition of a small amount of MeCN into the THF solution caused both a decrease in the formation of **1 b** and an increase in the formation of **2 b** to a similar degree. 2) In the presence of CO, both the disappearance of **1 a** and the formation of **2 b** were inhibited, but **1 b**, which did not form under an Ar atmosphere at all, was produced even in MeCN. If we take these results into consideration along with the finding that the formation of **2 b** was not dependent on the concentration of MeCN (>1 %) in the mixed solutions of THF–MeCN, we can conclude that the photochemical-induced loss of a CO ligand to give [Re(bpy)(CO)_2_Cl] is the initial step in the formation of both **1 b** and **2 c**, which is the precursor of **2 b**. For the production of either **1 b** or **2 c**, a flip of the Cl^−^ ligand of the initial intermediate(s) just after the dissociation of one of the CO ligands should be required [Eq. (4)]. The observed activation energy (11.1 kJ mol^−1^ in MeCN)^18^ for the formation of **2 b** is possibly attributable to this process.


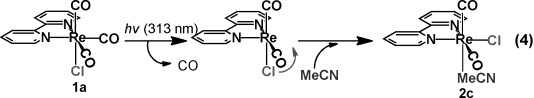
(4)

**Figure 3:**
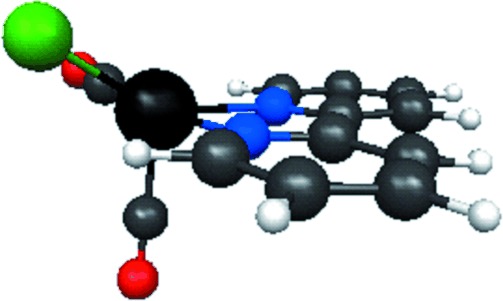
Optimized structure of the five-coordinate intermediates [Re(bpy)(CO)_2_Cl] calculated using the B3LYP/DFT method.

**Scheme 1:**
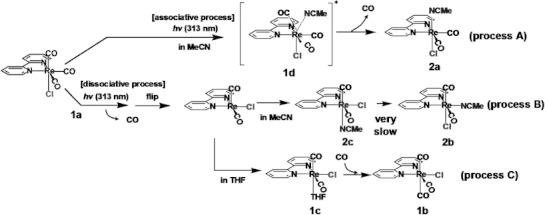
Mechanism of the photochemical reactions of **1 a** using 313 nm light in MeCN (processes A and B) or THF (process C).

The most stable structure of the coordinatively unsaturated intermediates [Re(bpy)(CO)_2_Cl] was calculated using the B3LYP/DFT method (Figure 3). In this structure, the Cl^−^ ligand is nearly coplanar with the bpy ligand, and the angle between the Cl–Re bond and the least-square plane of bpy is 36°. Therefore, it is understandable that the loss of CO from the excited state of **1 a** is followed by flip of the Cl^−^ ligand and then re-coordination of CO or coordination of MeCN to give **1 b** or **2 c**, respectively (processes C and B in Scheme 1).^19^ Slow isomerization of **2 c** gives the final product **2 b**.6a, 20

On the other hand, **2 a** should form by means of the associative mechanism initiated by the attack of an MeCN molecule on the excited state of **1 a** (process A in Scheme 1) because the yield of **2 a** was strongly dependent on the concentration of MeCN in the reaction solution, but the rate of formation of **2 a** was almost the same under a CO atmosphere as that under Ar.

We have recently reported that the photochemical formation of **2 a** and **2 b** from **1 a** in an MeCN solution with <330 nm light was completed within 50–100 ps after excitation, which was supported by the time-resolved IR spectroscopy data.9a As described later, time-resolved UV/Vis absorption spectroscopic results also indicate that the formation of **2 a** is complete within 100 ps. This is in striking contrast to the photochemical ligand substitution reaction of [W(phen)(CO)_4_] (phen=1,10-phenanthroline) with PR_3_ (R=Me, Ph, and so on), which requires a few nanoseconds for completion.^21^ The latter proceeds by means of the associative mechanism (i.e., nuclear attack of the phosphane on the lowest ^3^mLCT excited state of the complex), as shown in Equation (5), which is similar to the process used for the formation of **2 a**.


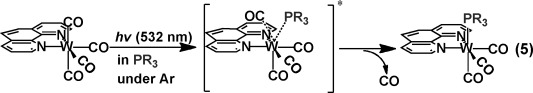
(5)

**Scheme 2:**
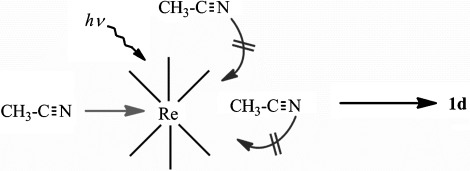
Formation of **1 d**.

To gain a deeper insight into the mechanism, we investigated the effects of the solvent viscosity on the photochemical reaction of **1 a** using a 1:1 mixture of *N*,*N*-dimethylformamide (the absolute viscosity coefficient at 298 K is 0.794 MPa s)–MeCN (0.369 MPa s) and *N*,*N*-dimethylacetamide (1.956 MPa s)–MeCN, which have similar relative permittivity values (*ε*_DMF_=36.7; *ε*_DMA_=37.8). Although the reaction rates in both mixed solutions decreased compared with that in neat MeCN because the concentration of MeCN was reduced to 50 %, **2 a** and **2 b** formed quantitatively in both instances, and their ratios of formation were almost the same as that observed in the 1:1 THF (0.456 MPa s)–MeCN mixed solution. The rates of the disappearance of **1 a** were also identical within experimental errors in the three mixed solvents at temperatures between 287 and 323 K (see Figure S4 in the Supporting Information). Therefore, we can conclude that the solvent viscosity does not affect the photochemical ligand substitution reaction, and it is clear that the formation of **2 a** does not include a process involving the diffusion of an MeCN molecule to the complex. The most reasonable mechanism for the formation of **2 a** is as follows: a MeCN molecule, which physically exists near an excited-state complex of **1 a**, the conformation of which is favorable for coordination to the Re center of the complex, rapidly coordinates to the complex without requiring any diffusion to give seven-coordinate intermediate **1 d** (Scheme 2). Subsequently, the CO ligand dissociates from **1 d** giving **2 a** (process A in Scheme 1). The photochemical-induced formation of **1 d** possibly proceeds by means of the attack of an MeCN molecule on the excited-state species of **1 a** with MLCT character, because in the MLCT excited state the electron density of the central Re is lower than that in the ground state. Therefore, nucleophilic attack by the nitrogen atom of MeCN at Re might become more favorable than attack at other excited states, such as MC and LC.

Dissociation of the CO ligand from seven-coordinate intermediate **1 d** apparently requires extra energy at this stage, in contrast to what is needed for ligand dissociation from the W complex. This is supported by the findings that the formation of **1 b** did not proceed by excitation with 366 nm light, which selectively produces the lowest and second-lowest ^1^mLCT excited states,8a and the quantum yield of the formation of **1 b** increased as the wavelength of irradiated light became shorter. On the other hand, photochemical ligand substitution of the W complex proceeds by irradiation even at 458–577 nm, which is the absorption region of the lowest ^1^mLCT band, and the quantum yield of the reaction was independent of the excitation wavelength in the range of 458–577 nm.^22^

**Identification and kinetics of the excited states using time-resolved spectroscopy**: For directly clarifying the dynamics of the photophysical processes of **1 a** and the chemical reactions of its reactive excited states and intermediates, ultrafast time-resolved UV-visible absorption (TR-UV/Vis), emission (TR-EM), and IR (TR-IR) spectroscopy was employed.

**Figure 4:**
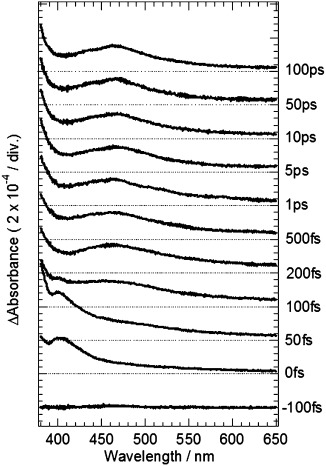
Transient absorption spectra of a solution of **1 a** in THF following excitation with a femtosecond 360 nm laser pulse.

Figure 4 shows the variation in the TR-UV/Vis of **1 a** in THF following excitation using a laser pulse of 360 nm. Excitation of **1 a** at 360–370 nm corresponds to the production of the ^1^mLCT excited states of **1 a**, and neither photochemical isomerization nor ligand substitution was induced by excitation in these regions, as described above. A relatively sharp absorption around 405 nm was observed just after excitation, which nearly disappeared within 100 fs, and was replaced by a broad absorption with a maximum at 460 nm. This absorption sharpened within 1 ps, and the maximum was slightly redshifted up to 475 nm and then slightly redshifted again within several tens of picoseconds. After this, no significant change in the absorption was observed up to 200 ps.

**Figure 5:**
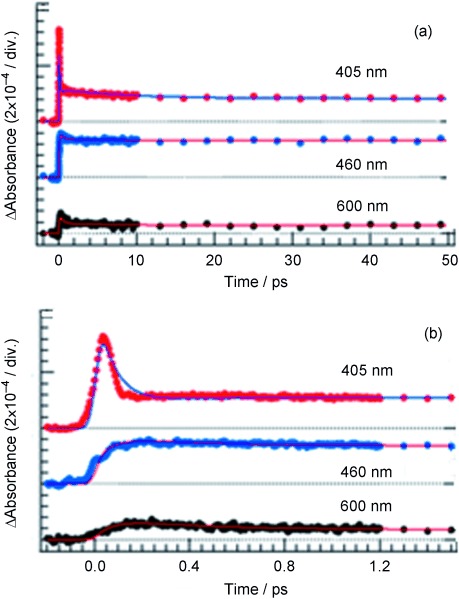
Time profiles of the transient absorbance of a solution of **1 a** in THF following excitation with a femtosecond 360 nm laser pulse. The monitoring wavelengths are at 405, 460, and 600 nm. Solid lines are curves calculated from a triple-exponential function. Coherent oscillations in the time profiles were not taken into account in the analysis.

Time-profiles of the absorption at 405, 460, and 600 nm, which can be totally fitted with triple-exponential functions with lifetimes of 70 fs, 380 fs, and 9.1 ps, respectively, are shown in Figure 5.

**Figure 6:**
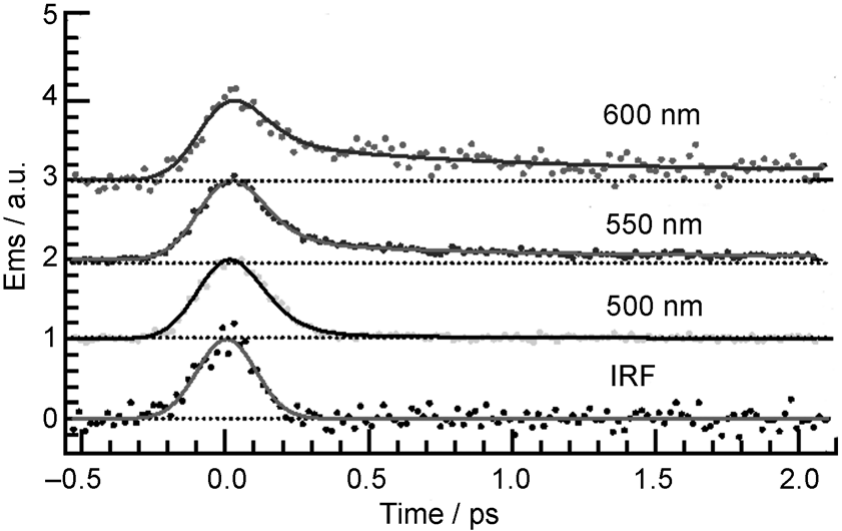
Time profiles of the emission up-conversion signals of a solution of **1 a** in THF following excitation with a femtosecond 400 nm laser pulse. The monitoring wavelengths are 600, 550, and 500 nm. Solid lines are results calculated from a double-exponential function and residual signal.

Figure 6 shows emission-decay curves from **1 a** after the laser flash (excitation wavelength: 400 nm). All the emission-decay profiles could be reproduced by a double-exponential function with time constants of (71±10) and (360±50) fs. In addition, the residual signal remained even after 2 ps. With a decrease in the monitoring wavelength, the contribution from the fast decaying component is pronounced, and the contribution from the residual signal becomes small. Vlček and co-workers have also reported femtosecond-emission spectroscopic results for **1 a** in MeCN, and on the basis of DFT calculations, they proposed that irradiation using 400 nm light induces the ^1^mLCT excited state with A′ symmetry, from which intersystem crossing occurs with *τ*=(85±8) fs to produce ^3^LC and ^3^mLCT, which is still vibrationally hot (^3^mLCT(HV)).8a, e, f Internal conversion of the ^3^LC state to the ^3^mLCT(HV) state with A′′ symmetry proceeds with *τ*=(340±50) fs.

**Figure 7:**
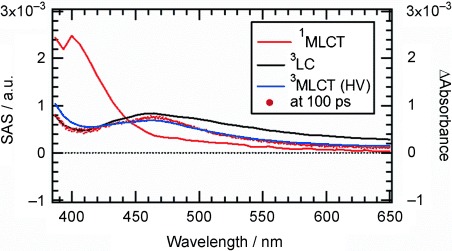
Species-associated spectra and transient absorption spectrum of a solution of **1 a** in THF following excitation with a femtosecond 360 nm laser pulse.

These observations and information clearly indicate that the absorption at 405 nm can be attributed to the ^1^mLCT excited state, from which fluorescence is observed at a shorter wavelength than phosphorescence from the corresponding triplet excited state, and that intersystem crossing to the ^3^LC and ^3^mLCT(HV) excited states occurs with a lifetime of 70–80 fs in THF. Internal conversion from ^3^LC to ^3^mLCT(HV) and spectral evolution by means of a vibrational relaxation process of ^3^mLCT(HV) proceed with lifetimes of approximately 370 fs and 9.1 ps, respectively, giving the lowest vibrational ^3^mLCT excited states (^3^mLCT(LV)). To obtain the spectrum corresponding to each of the time constants, a global analysis was applied, the result of which is shown in Figure 7. In this figure, the spectrum at 100 ps after excitation, which is attributed to ^3^mLCT(LV),8c is also listed.

**Figure 8:**
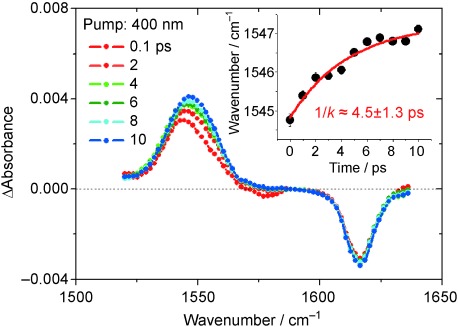
TR-IR spectra of a solution of **1 a** in THF using an excitation wavelength of 400 nm. The inset shows the center wavenumber of the absorption peak as a function of the delay time.

**Figure 9:**
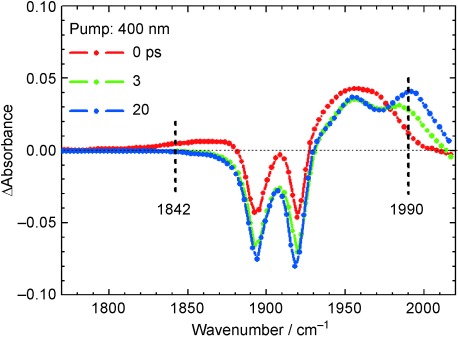
TR-IR spectra of a solution of **1 a** in THF at 0, 3, and 20 ps using a 400 nm laser pulse for excitation.

**Figure 10:**
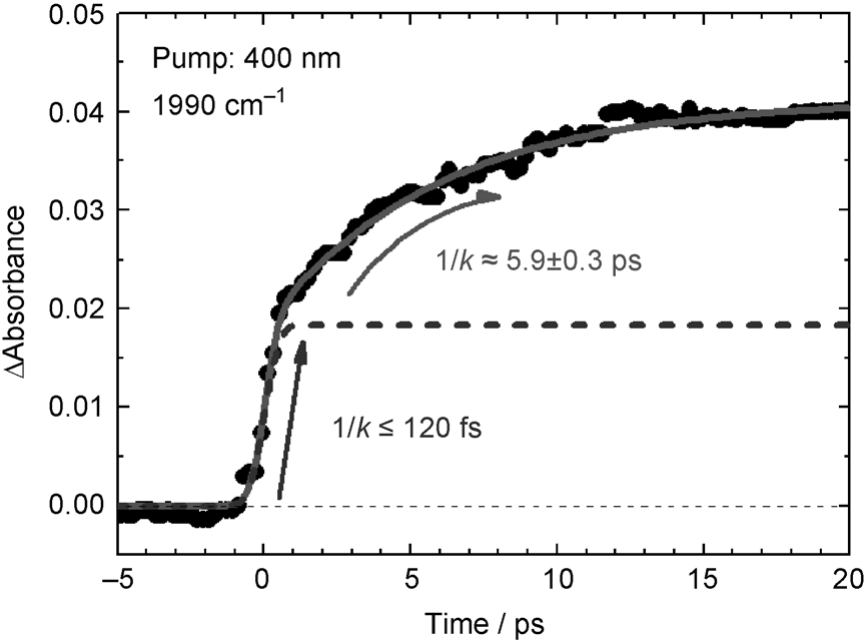
Time profile of the absorbance changes for a solution of **1 a** in THF following excitation with a 400 nm laser pulse. The monitoring wavenumber is 1990 cm^−1^. The solid line is a curve calculated from a single-exponential and step function convoluted with a laser function.

These relaxation processes of the ^3^LC and ^3^mLCT(HV) excited states were also observed by time-resolved infrared (TR-IR) spectroscopy. Just after laser irradiation (400 nm, full width at half-maximum (fwhm)=120 fs), a new absorption and a negative peak were observed at 1545 and 1616 cm^−1^, respectively, attributable to the vibrational stretching bands of the bpy ligand (Figure 8). Although the shape and strength of the negative peak did not change within 10 ps, the center of the positive peak, determined by a Lorentz-type peak fitting analysis, was blueshifted up to 1547 cm^−1^ with 1/*k*=(4.5±1.3) ps (inset of Figure 8). A very broad absorption between 1830 and 1870 cm^−1^ and an absorption with a maximum at 1950 cm^−1^ were also observed along with negative peaks at 1893 and 1917 cm^−1^, which are attributable to a decrease of the CO stretching bands in the ground state of **1 a** just after the laser flash (Figure 9). The absorption at 1950 cm^−1^, attributable to the ^3^mLCT excited state of **1 a**,1f sharpened and split into two peaks with maxima at 1955 and 1991 cm^−1^, which are attributable to two of three CO stretching bands of ^3^mLCT(LV),1f with 1/*k*=(5.9±0.3) ps (Figure 10). These changes likely result from the relaxation of ^3^mLCT(HV) to ^3^mLCT(LV). It should be noted that the time constant of the temporal evolution of the electronic spectra resulting from vibrational cooling does not directly correspond to the dissipation process of excess vibrational energy.^23^ That is, the temperature dependence of the electronic absorption spectra, which is inherent to the individual system, impacts the time evolution of these electronic absorption spectra through vibrational cooling. Accordingly, the time constant for the spectral evolution resulting from vibrational cooling is dependent on the detection methods, although the timescale for the changes is consistent between spectra. Therefore, in this case, the time constant for the dissipation of excess energy from ^3^mLCT(HV) should be in the time range of 4–10 ps.

**Figure 11:**
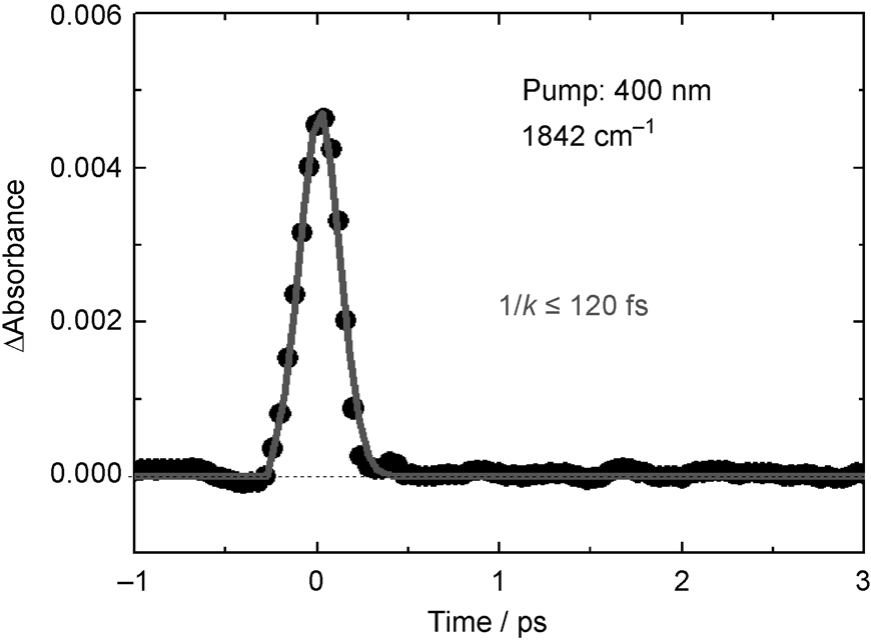
Time profile of the absorbance changes for a solution of **1 a** in THF following excitation with a 400 nm laser pulse. The monitoring wavenumber is 1842 cm^−1^. The solid line is a curve calculated from a single-exponential function convoluted with a laser function.

**Figure 12:**
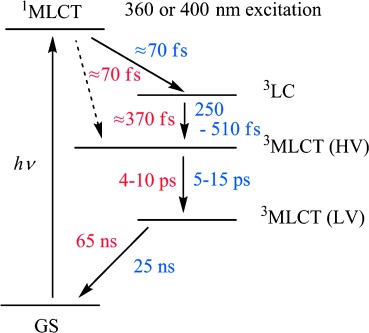
Photophysical processes of **1 a** following excitation at ≥360 nm in THF (lifetimes in red) and MeCN (lifetimes in blue). The lifetimes of the ^3^mLCT(LV) are reported values.1d, h

The results in Figure 10 also indicate that there was a faster rise of the peak before the relaxation process. This is further supported by the rise and decay profile at 1842 cm^−1^ (Figure 11). This feature might be attributable to intersystem crossing from ^1^mLCT because these phenomena occurred within the time frame of a laser pulse (fwhm=120 fs). The photophysics of **1 a** following excitation at the wavelength of 360 or 400 nm is summarized in Figure 12.

**Figure 13:**
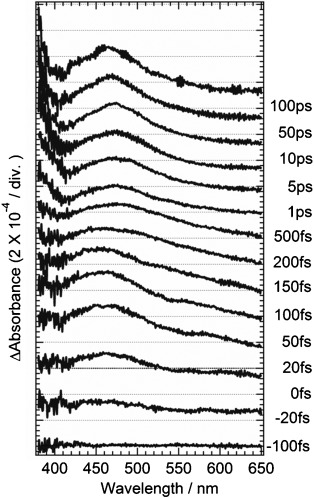
Transient absorption spectra of a solution of **1 a** in THF following excitation with a femtosecond 270 nm laser pulse.

**Figure 14:**
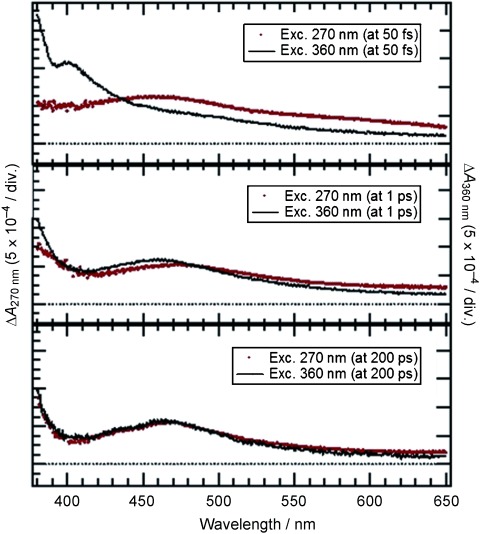
A comparison of the transient absorption spectra of a solution of **1 a** in THF obtained using laser light between 270 and 360 nm.

Excitation using a laser pulse of ≤270 nm caused TR-spectral changes that were very different from those resulting from a ≥360 nm pulse, as described above. The transient UV-visible absorption spectra are shown in Figure 13. Figure 14 shows a comparison of the spectra obtained using 270 and 360 nm laser light, which were observed at 50 fs, 1 ps, and 200 ps after laser excitation. The relatively sharp absorption at 405 nm observed at 50 fs following 360 nm excitation, which is attributed to the ^1^mLCT excited state, was not detected following 270 nm excitation at any time up to 200 ps. Instead, a very broad band at 460 nm was observed with a shoulder near 560 nm (Figure 14, 50 fs). This differing spectral behavior continued for several picoseconds after the laser flash (for example, see Figure 14, 1 ps). However, the spectra observed at and after 10 ps following excitation were almost identical to those observed using 360 nm excitation (Figure 14, 200 ps), which are attributable to the ^3^mLCT(LV) excited state.

**Figure 15:**
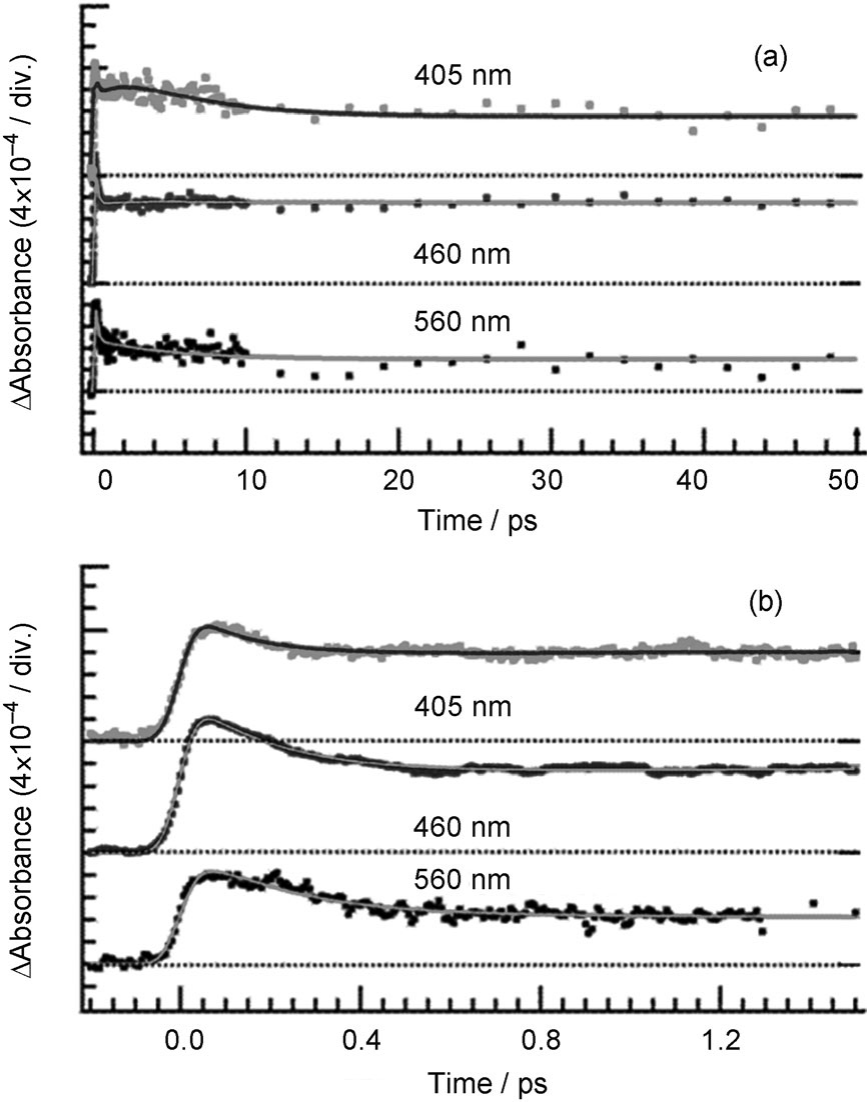
Time profiles of the transient absorbance of a solution of **1 a** in THF following excitation with a femtosecond 270 nm laser pulse. The monitoring wavelengths are at 405, 460, and 560 nm. Solid lines are curves calculated from a triple-exponential function with lifetimes of 25 fs, 200 fs, and 4.9 ps.

Figure 15 shows the time profiles of the absorbance at 405, 460, and 560 nm after excitation using a 270 nm laser. Triple-exponential functions were employed to analyze the time profiles, from which lifetimes were estimated to be 25 fs, 200 fs, and 5–10 ps. The faster time constant is less reliable because this time constant is much shorter than the fwhm of the cross correlation at the sample position. The iterative analysis, however, indicated that a much shorter time constant was necessary to reproduce the experimental results. The longest time constant may be safely ascribed to vibrational cooling.

**Figure 16:**
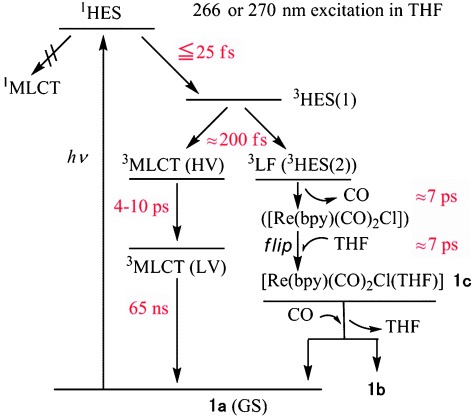
Photophysical and photochemical processes of **1 a** in THF following excitation with ≤270 nm light. The red text shows the lifetimes.

As shown above, the spectra and their time evolution observed following 270 nm pulsed excitation were quite different from those obtained using 360 nm excitation. These results strongly indicate the following photophysical and photochemical processes (Figure 16):

1. Intersystem crossing from singlet higher excited states (^1^HES), which are produced by excitation at 270 nm, to the triplet reactive excited state(s) (^3^HES(1)) was much faster than intersystem crossing from ^1^mLCT to ^3^mLCT observed following 360 nm excitation, and ^1^mLCT was not observed following excitation at 270 nm.^24^
2. ^3^HES(1) decayed with a lifetime of approximately 200 fs.
3. The decay of ^3^HES(1) produces not only ^3^mLCT(HV), which is still vibrationally hot, but also the other excited state(s) (^3^HES(2)) with broad absorption around 570 nm. For these processes, we could extract only one decay lifetime of approximately 200 fs. This indicates that these two decay processes proceed on a similar timescale. We will discuss the identification of ^3^HES(2) later.
4. After the decay of ^3^mLCT(HV) and ^3^HES(2), the remaining excited state is ^3^mLCT, which has already been vibrationally cooled (i.e., ^3^mLCT(LV)).

**Figure 17:**
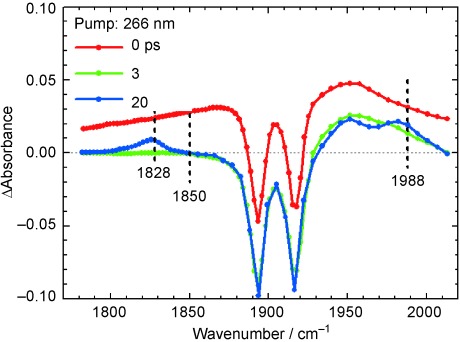
TR-IR spectra of a solution of **1 a** in THF at 0, 3, and 20 ps following excitation by a 266 nm laser pulse.

**Figure 18:**
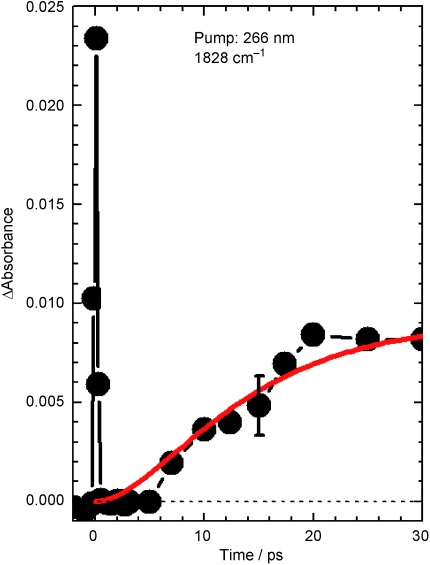
Time profile of the absorbance changes of a solution of **1 a** in THF at 1828 cm^−1^. The red solid line is derived from solving the rate equation of the two-step reaction with 1/*k*_1_≍7 ps and 1/*k*_2_≍7 ps.

The TR-IR spectra obtained by excitation with a 266 nm laser pulse (fwhm=120 fs) gave additional useful information about the intermediates in the photoisomerization of **1 a** (Figure 17). Compared with that observed by using 400 nm excitation (Figure 9), a broader absorption ranging from 1750 to over 2000 cm^−1^ was noted in this case just after the laser pulse, which then decayed within a laser pulse (see Figure S5 in the Supporting Information: absorption at 1850 cm^−1^). This process is attributable to the formation of ^3^HES(1) because of the similar timescale (≤25 fs). After an induction period of several picoseconds, a new absorption appeared at approximately 1828 cm^−1^ (Figure 17) with a rise time of 1/*k*=(7±2) ps (Figure 18). This relatively sharp absorption did not change for up to 1 ns. This can be attributed to the unsymmetrical CO vibrational band of the bis-carbonyl–Re^I^ complex [Re(bpy)(CO)_2_Cl(thf)] (**1 c**), which should be produced by substitution of one of the CO ligands with a solvent molecule, as suggested by comparison with the IR spectra of both (*OC*-6-34)- and (*OC*-6-44)-[Re(bpy)(CO)_2_Cl(MeCN)] (**2 a** and **2 b**), which show similar absorptions at 1832 and 1830 cm^−1^, respectively. The induction period for the formation of **1 c** strongly suggests that there is another short-lived species that is produced from ^3^HES(2) for several picoseconds and then converted into **1 c** over another several picoseconds. It is possibly the coordinatively unsaturated complex [Re(bpy)(CO)_2_Cl], seeing as the formation of **1 c** proceeds by the dissociative mechanism as described in the previous section. It is also logically concluded that ^3^HES(2) is the excited state with antibonding character such as the triplet ligand field (^3^LF) state.^25^ Intermediate **1 c** reacts with the detached CO to give the product *mer*-[Re(bpy)(CO)_3_Cl] (**1 b**) or to return to starting complex **1 a**.^26^ The absorption between 1930 and 2010 cm^−1^ corresponds to the 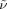
_CO_ region of tricarbonyl complexes with a Re center that is more electron poor than the ground state of the corresponding Re^I^–tricarbonyl complexes, such as ^3^mLCT. This absorption, resulting from 266 nm excitation (Figure 17), was different from that obtained using 400 nm excitation just after laser irradiation (Figure 9), but after a few picoseconds both absorptions were similar. The rates of these processes following 266 nm excitation were 1/*k*≤120 fs and 1/*k*=(4.4±0.6) ps, respectively (see Figure S6 in the Supporting Information), which are in good agreement with those for the intersystem crossing from ^1^HES and spectral evolution by the vibrational relaxation process of ^3^mLCT(HV) to ^3^mLCT(LV), both obtained by TR-UV/Vis (≤25 fs, 5–10 ps). The following spectral changes in this region were similar between the 266 and 400 nm excitation runs.

Although ^3^LC might also form from ^3^HES(1), we do not have clear evidence to support this because the spectra of both ^3^LC and ^3^HES(1) were very broad and were observed at similar UV-visible and IR regions.

**Figure 19:**
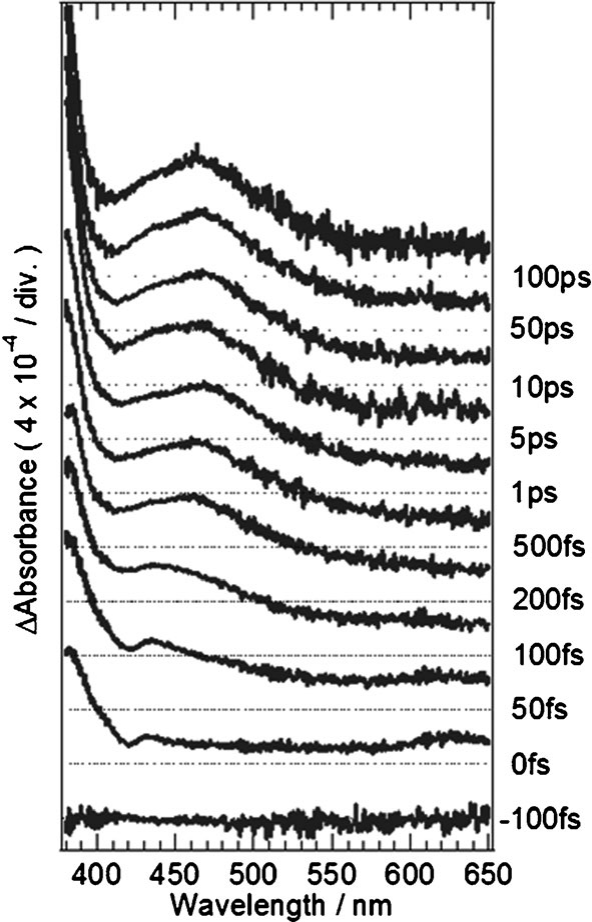
Transient UV-visible absorption spectra of a solution of **1 a** in MeCN following excitation with a femtosecond 370 nm laser pulse.

In a solution in MeCN, time-resolved UV-visible absorption spectra (Figure 19) showed a temporal evolution similar to that observed using 360 nm laser excitation in THF (Figure 4), with the exception of the UV-visible absorption spectrum of the ^1^mLCT excited state, which showed a maximum at 430 nm and a 25 nm redshift relative to that in THF. The lifetime of the ^1^mLCT was (72±2) fs (Figure S7), which is consistent with TR-emission measurements (*τ*_em_=(71±2) fs, observed by us, Figure S9; (85±8) fs, reported by Vlček et al.8a). The transient profiles and the lifetimes of the ^1^mLCT, ^3^LC, and ^3^mLCT(HV) are shown in Figure S7 in the Supporting Information, and their lifetimes are summarized in Figure 12.

**Figure 20:**
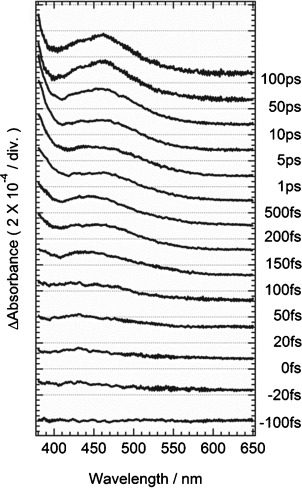
Transient absorption spectra of a solution of **1 a** in MeCN following excitation with a femtosecond 270 nm laser pulse.

The time evolution of the transient UV-visible absorption spectra of **1** in MeCN excited with a 270 nm femtosecond laser pulse was also quite different from that resulting from 370 nm laser excitation (Figure 19). As shown in Figure 20, transient spectra immediately after the 270 nm excitation show no sharp peak at 430 nm ascribable to ^1^mLCT. However, a very broad absorption band with increased intensity in the shorter wavelength region was observed immediately after excitation. In the time range of <100 fs, a new absorption band in the 430–450 nm region gradually appeared, and then the absorption spectrum due to ^3^mLCT was mainly confirmed at and after several picoseconds following excitation. Time profiles of the transient absorbance at several wavelength points are exhibited in Figure S8 in the Supporting Information, in which all of the time profiles could be reproduced by a triple-exponential function with time constants of 30 fs, 130 fs, and 5–15 ps. As was mentioned in the previous sections, the faster time constant was less reliable because of the rather long cross-correlation width of the pump and probe pulses.

**Figure 21:**
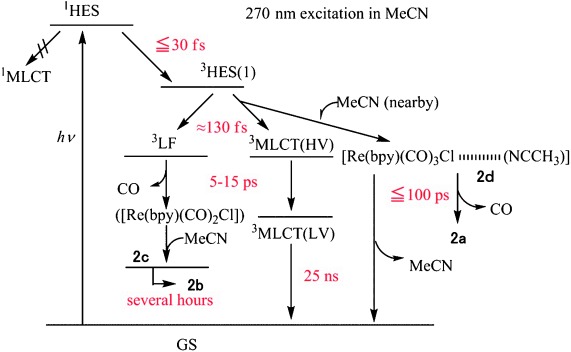
Photophysical and photochemical processes of a **1 a** in MeCN following excitation with 270 nm light. The red text shows the lifetimes.

From the above experimental results, the photophysics and photochemistry of **1 a** excited with 270 nm light in MeCN can be summarized (Figure 21). In addition to the 30 fs decay, which is attributable to intersystem crossing, two rise and/or decay components were observed, the lifetimes of which were approximately 130 fs and 5–15 ps. Because the component with *τ*≍130 fs was detected only under the conditions that included both the MeCN solvent and 270 nm excitation wavelength, and because it was faster than the corresponding component measured in THF (ca. 200 fs), it can be attributed to the process for the formation of the intermediate of **2 a** (i.e., [Re(bpy)(CO)_3_Cl⋅⋅⋅(NCCH_3_)], **2 d**) from ^3^HES(1). This component (*τ*≍130 fs) should also be involved in the other conversions starting from ^3^HES(1) (i.e., the formation of ^3^mLCT(HV) and ^3^LF). The additional pass to produce **2 a** can be induced only in an MeCN solution or mixed solutions of THF with a high concentration of MeCN, but did not occur in THF alone. Since the yield for the formation of **2 c** was not affected by the concentration of MeCN in the reaction solution, the process for the formation of **2 d** can compete with that for the formation of ^3^mLCT(HV) but not with that for the formation of ^3^LF. Because the lifetime of the ^3^HEM(1) state should be too short for bulk MeCN molecules to diffuse and collide with this excited state existing in a suitable conformation for addition to the Re center, only MeCN molecules located near the excited complex, conveniently having the appropriate geometry, should be able to interact with the central Re (Scheme 2). It is noteworthy that this consideration of the timescale is very consistent with that of the solvent effects described in the “Mechanisms of the photochemical reactions”. We have already reported the TR-IR spectra of **1 a** in MeCN after 266 nm (fwhm=150 fs) excitation, and they clearly indicated that the formation of the ligand-substitution products, **2 a** and **2 c**, was already complete within 100 ps after excitation.9a Therefore, the decay of **2 d** by the elimination of the CO ligand leading to **2 a** and probably the recovery of **1 a** by the elimination of MeCN might proceed within several tens of picoseconds.

The component with *τ*=5–15 ps is attributable to the decays of both ^3^mLCT(HV) and ^3^LF. As described in the “Mechanisms of the photochemical reactions” section, the formation of **2 c** in MeCN, which isomerizes to **2 b** with a lifetime of several hours, proceeds through the same intermediate that leads to **1 b** in THF (i.e., the coordinatively unsaturated complex [Re(bpy)(CO)_2_Cl]).

## Conclusion

The mechanisms of the photochemical reactions of **1 a** have been clarified in detail by the combination of steady-state irradiation, TR-UV/Vis, TR-IR, and TR-EM experiments. Irradiation of **1 a** using high-energy light (270 nm) induces the formation of the higher singlet excited state (^1^HES: *τ*≤30 fs), which is quickly converted to the corresponding triplet state (^3^HES(1)) without the formation of ^1^mLCT. In a solution in THF, ^3^HES(1) is competitively converted to both ^3^LF and ^3^mLCT excited states with 1/*k*≍200 fs. The former is a reactive state giving coordinatively unsaturated [Re(bpy)(CO)_2_Cl] and then THF complex **1 c**, both with 1/*k*≍7 ps. Re-coordination of CO to **1 c** gives both starting complex **1 a** and meridional isomer **1 b**. In a solution in MeCN, irradiation of **1 a** using high-energy light also gives the coordinatively unsaturated complex [Re(bpy)(CO)_2_Cl] through ^3^LF, which is rapidly converted to one of the MeCN complexes (**2 c**). This process requires thermal activation (*E*_a_=(1112±58) cm^−1^) probably because a flip of the Cl^−^ ligand is necessary. The isomerization of **2 c** to **2 b** proceeds within several hours even at ambient temperature. In addition, another ligand-substitution process occurs in MeCN. Seven-coordinate complex **1 d** is produced with 1/*k*≍130 fs and is then converted to **2 a** within 100 ps. However, irradiation of **1 a** using lower energy light (360 nm) does not cause any photochemical reactions but gives the ^1^mLCT excited state, which demonstrated a relatively sharp absorption band at 405 nm in THF and at 430 nm in MeCN. Intersystem crossing from the ^1^mLCT excited state with a lifetime of approximately 70 fs gives both ^3^LC and ^3^mLCT(HV). Key processes could be followed by transient UV-visible absorption, emission, and IR spectroscopy, that is, the internal conversion process from ^3^LC to ^3^mLCT(HV) (1/*k*=several hundreds of femtoseconds) and the vibrational relaxation process from ^3^mLCT(HV) to the lowest excited state ^3^mLCT(LV) (1/*k*=several picoseconds).

These results also give important information on the limitation of **1 a** as a photofunctional molecule under irradiation condition with >330 nm light. Even in solution with low coordination abilities, the very rapid deactivation process proceeds by the associative mechanism. The rapid photochemical ligand-substitution reaction by the dissociative mechanism might be also a problem. Therefore, excitation of **1 a** to the HES should be avoided for use of **1 a** as a photofunctional molecule.

## Supplementary material

Detailed facts of importance to specialist readers are published as ”Supporting Information”. Such documents are peer-reviewed, but not copy-edited or typeset. They are made available as submitted by the authors.

## References

[1a] Wrighton M, Morse DL (1974). J. Am. Chem. Soc.

[1b] Luong JC, Falynak H, Wrighton MS (1979). J. Am. Chem. Soc.

[1c] Giordano PJ, Wrighton MS (1979). J. Am. Chem. Soc.

[1d] Worl LA, Duesing R, Chen P, Ciana LD, Meyer TJ (1991). J. Chem. Soc. Dalton Trans.

[1e] Striplin DR, Crosby GA (1994). Chem. Phys. Lett.

[1f] Clark IP, George MW, Johnson FPA, Turner JJ (1996). Chem. Commun.

[1g] Vanhelmont FWM, Hupp JT (2000). Inorg. Chem.

[1h] Stufkens DJ, Vlček A (1998). Coord. Chem. Rev.

[1i] Vlček A (2010). Top. Organometal. Chem.

[1j] Rossenaar BD, Stufkens DJ, Vlček A (1996). Inorg. Chem.

[2a] Hawecker J, Lehn JM, Ziessel R (1986). Helv. Chim. Acta.

[2b] Kalyanasundaram K (1982). Coord. Chem. Rev.

[2c] Kutal C, Weber MA, Ferraudi G, Geiger D (1985). Organometallics.

[2d] Kutal C, Corbin AJ, Ferraudi G (1987). Organometallics.

[2e] Kurz P, Probst B, Spingler B, Alberto R (2006). Eur. J. Inorg. Chem.

[2f] Takeda H, Koike K, Inoue H, Ishitani O (2008). J. Am. Chem. Soc.

[2g] Gholamkhass B, Mametsuka H, Koike K, Furue M, Ishitani O (2005). Inorg. Chem.

[2h] Koike K, Naito S, Sato S, Tamaki Y, Ishitani O (2009). J. Photochem. Photobiol. A.

[2i] Takeda H, Ishitani O (2010). Coord. Chem. Rev.

[2j] Doherty MD, Grills DC, Muckerman JT, Polyansky DE, Fujita E (2010). Coord. Chem. Rev.

[3] Yam VW-W, Li B, Yang Y, Chu BWK, Wong KMC, Cheung KK (2003). Eur. J. Inorg. Chem.

[4] Polo AS, Itokazu MK, Murakami Iha NY (2004). Coord. Chem. Rev.

[5a] Bélanger S, Hupp JT, Stern CL, Slone RV, Watson DF, Carrell TG (1999). J. Am. Chem. Soc.

[5b] Sun SS, Lees AJ (2000). J. Am. Chem. Soc.

[5c] Yam VW-W, Lo W-Y, Lam C-H, Fung WK-M, Wong KM-C, Lau VC-Y, Zhu N (2003). Coord. Chem. Rev.

[5d] Kelley RF, Lee SJ, Wilson TM, Nakamura Y, Tiede DM, Osuka A, Hupp JT, Wasielewski MR (2008). J. Am. Chem. Soc.

[5e] Yamamoto Y, Sawa S, Funada Y, Morimoto T, Falkenstrom M, Miyasaka H, Shishido S, Ozeki T, Koike K, Ishitani O (2008). J. Am. Chem. Soc.

[5f] Lo W-Y (2010). Top. Organomet. Chem.

[5g] Yamamoto Y, Tamaki Y, Yui T, Koike K, Ishitani O (2010). J. Am. Chem. Soc.

[6] Koike K, Okoshi N, Hori H, Takeuchi K, Ishitani O, Tsubaki H, Sakamoto K, Clark IP, George MW, Johnson FPA, Turner JJ (2002). J. Am. Chem. Soc.

[7a] Smothers WK, Wrighton MS (1983). J. Am. Chem. Soc.

[7b] Bredenbeck J, Helbing J, Hamm P (2004). J. Am. Chem. Soc.

[8a] Cannizzo A, Blanco-Rodríguez AM, Nahhas A, Šebera J, ZáliŠ S, Vlček A, Chergui M (2008). J. Am. Chem. Soc.

[8b] Nahhas AE, Cannizzo A, Mouril FV, Blanco-Rodríguez AM, ZáliŠ S, Vlček A, Chergui M (2010). J. Phys. Chem. A.

[8c] Liard DJ, Busby M, Matousek P, Towrie M, Vlček A (2004). J. Phys. Chem. A.

[8d] Dattelbaum DM, Omberg KM, Schoonover JR, Martin RL, Meyer TJ (2002). Inorg. Chem.

[8e] El Nahhas A, Consani C, Blanco-Rodríguez AMa, Lancaster KM, Braem O, Cannizzo A, Towrie M, Clark IP, ZáliŠ S, Chergui M, Vlček A (2011). Inorg. Chem.

[8f] Ko C-C, Kwok W-M, Yam VW-W, Phillips DL (2006). Chem. Eur. J.

[8g] Damrauer NH, Cerullo G, Yeh A, Boussie TR, Shank CV, McCusker JK (1997). Science.

[9a] Sato S, Sekine A, Ohashi Y, Ishitani O, Blanco-Rodríguez AM, Vlček A, Unno T, Koike K (2007). Inorg. Chem.

[9b] Sato S, Morimoto T, Ishitani O (2007). Inorg. Chem.

[10a] Becke AD (1988). Phys. Rev. A.

[10b] Lee C, Yang W, Parr RG (1988). Phys. Rev. B.

[10c] Miehlich B, Savin A, Stoll H, Preuss H (1989). Chem. Phys. Lett.

[11] Yang L, Ren AM, Feng JK, Liu XJ, Ma YG, Zhang M, Liu XD, Shen JC, Zhang HX (2004). J. Phys. Chem. A.

[12a] http://classic.chem.msu.su/gran/firefly/index.html.

[12b] Schmidt MW, Baldridge KK, Boatz JA, Elbert ST, Gordon MS, Jensen JH, Koseki S, Matsunaga N, Nguyen KA, Su SJ, Windus TL, Dupuis M, Montgomery JA (1993). J. Comput. Chem.

[13a] Hay PJ, Wadt WR (1985). J. Chem. Phys.

[13b] Wadt WR, Hay PJ (1985). J. Chem. Phys.

[14a] Ditchfield R, Hehre WJ, Pople JA (1971). J. Chem. Phys.

[14b] Hariharan PC, Pople JA (1973). Theor. Chim. Acta.

[14c] Hehre WJ, Ditchfield R, Pople JA (1972). J. Chem. Phys.

[15a] Chosrowjan H, Mataga N, Nakashima N, Imamoto Y, Tokunaga F (1997). Chem. Phys. Lett.

[15b] Chosrowjan H, Taniguchi S, Mataga N, Unno M, Yamauchi S, Hamada N, Kumauchi M, Tokunaga F (2004). J. Phys. Chem. B.

[16a] Murakami M, Miyasaka H, Okada T, Kobatake S, Irie M (2004). J. Am. Chem. Soc.

[16b] Katayama T, Ishibashi Y, Morii Y, Ley C, Brazard J, Lacombat F, Plaza P, Martin MM, Miyasaka H (2010). Phys. Chem. Chem. Phys.

[16c] Ishibashi Y, Katayama T, Ota C, Kobatake S, Irie M, Yokoyama Y, Miyasaka H (2009). New J. Chem.

[17] Matsubara Y, Okimoto Y, Yoshida T, Ishikawa T, Koshihara S, Onda K (2011). J. Phys. Soc. Jpn.

[19a] Rosa A, Ricciardi G, Baerends EJ, Stufkens DJ (1996). J. Phys. Chem.

[19b] Vlček A, Farrell IR, Liard DJ, Matousek P, Towrie M, Parker AW, Grills DC, George MW (2002). J. Chem. Soc. Dalton Trans.

[21a] Lindsay E, Vlček A, Langford CH (1993). Inorg. Chem.

[21b] Vlček A (1998). Coord. Chem. Rev.

[22] van Dijk HK, Servaas PC, Stufkens DJ, Oskam A (1985). Inorg. Chim. Acta.

[23] Miyasaka H, Hagihara M, Okada T, Mataga N (1992). Chem. Phys. Lett.

[24] Baková R, Chergui M, Daniel C, Jr AVlček, Zális S (2011). Coord. Chem. Rev.

[25] Záliš S, Busby M, Kotrba T, Matousek P, Towrie M, Vlček A (2004). Inorg. Chem.

