# Supplementary material for: Photochemistry of fac-[Re(bpy)(CO)3Cl]
Source: Chemistry. 2012 Oct 18;18(49):15722–34. doi: 10.1002/chem.201202734 (PMC3546374; doi:10.1002/chem.201202734)
Supplement: Supplementary file 1 [file chem0018-15722-SD1.pdf]

CHEMISTRY
A EUROPEAN JOURNAL

Supporting Information

© Copyright Wiley-VCH Verlag GmbH & Co. KGaA, 69451 Weinheim, 2012

Photochemistry of *fac*-[Re(bpy)$(CO)_3$Cl]**

Shunsuke Sato,[a, g] Yasuo Matubara,[a, g] Kazuhide Koike,[b, e] Magnus Falkenström,[d] Tetsuro Katayama,[d, e] Yukihide Ishibashi,[d, e] Hiroshi Miyasaka,*[d, e] Seiji Taniguchi,[c] Haik Chosrowjan,[c] Noboru Mataga†,[c] Naoto Fukazawa,[a] Shinya Koshihara,[a, e] Ken Onda,*[f, g] and Osamu Ishitani*[a, e, h]

chem_201202734_sm_miscellaneous_information.pdf

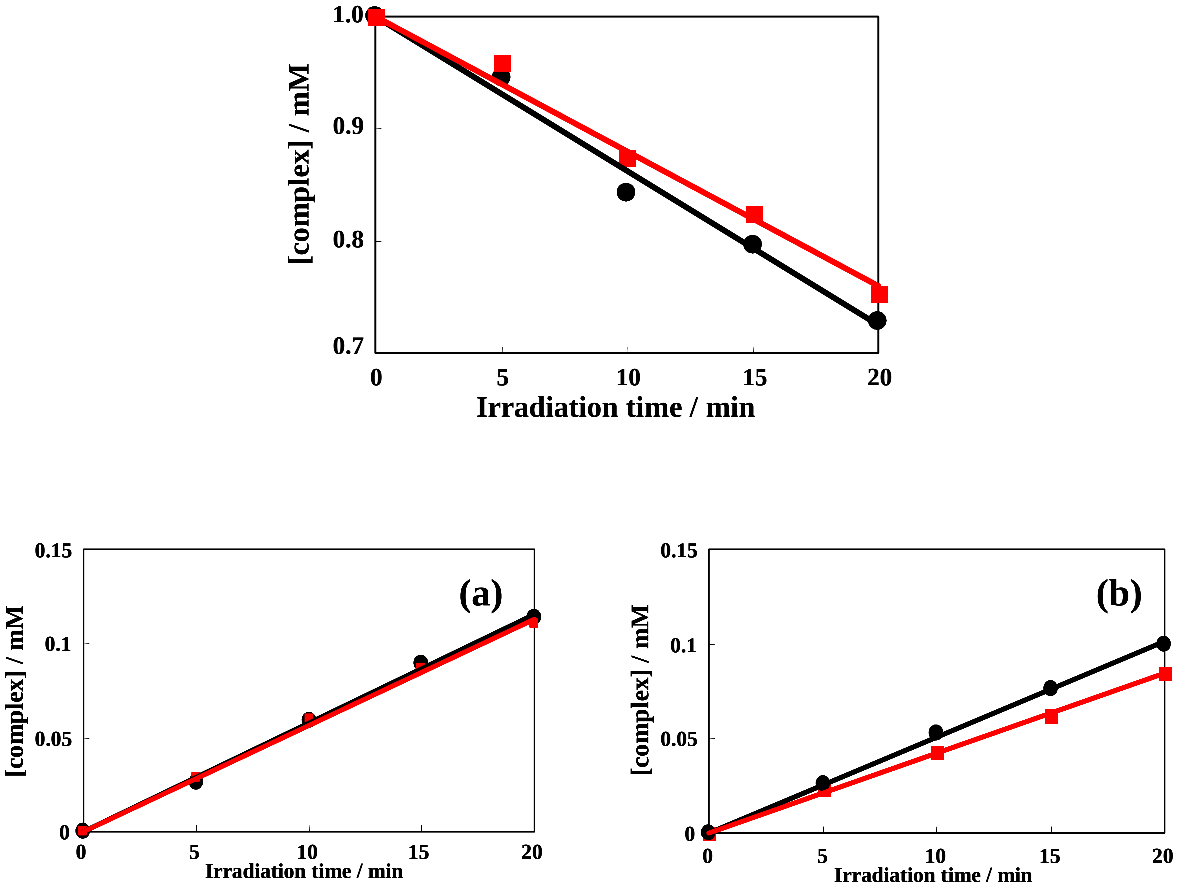

Figure S1. Decrease of **1a** (a) and formation of **2a** (b) and **2b** (c) by irradiation to an MeCN solution containing **1a** (1 mM) using 313-nm light under Ar (black) or CO (red) atmosphere.

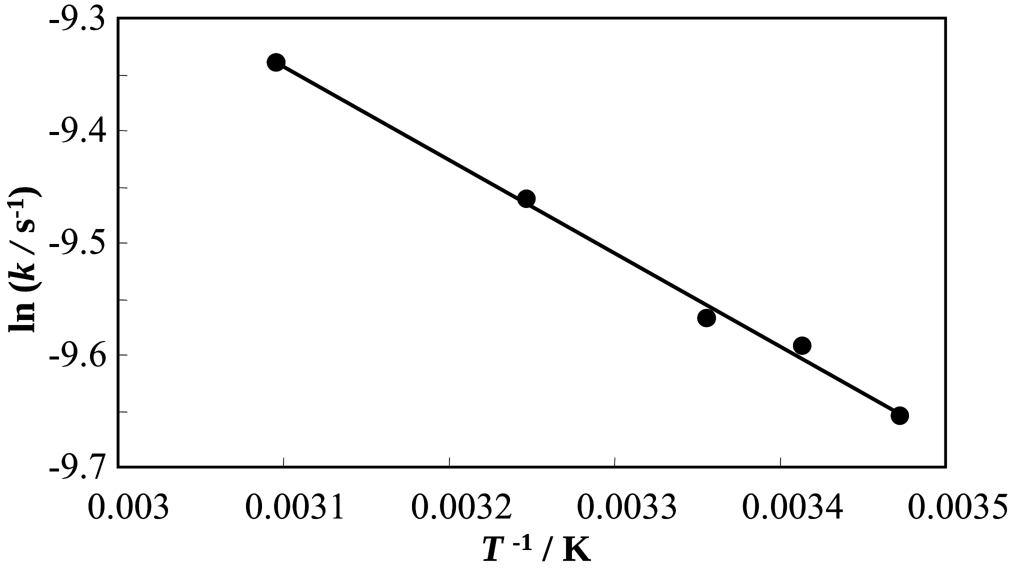

Figure S2 Arrhenius plots for decrease of **1a** by irradiation using 313-nm light in an MeCN solution containing 1.0 mM of **1a** under an Ar atmosphere.

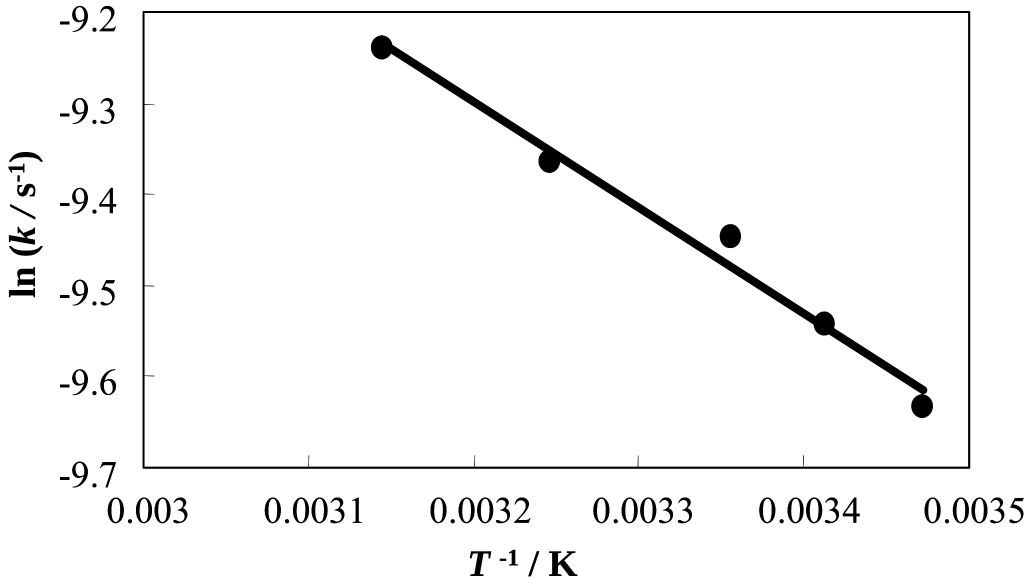

Figure S3 Arrhenius plots for decrease of **1a** by irradiation using 313-nm light in a THF solution containing 1.0 mM of **1a** under an Ar atmosphere.

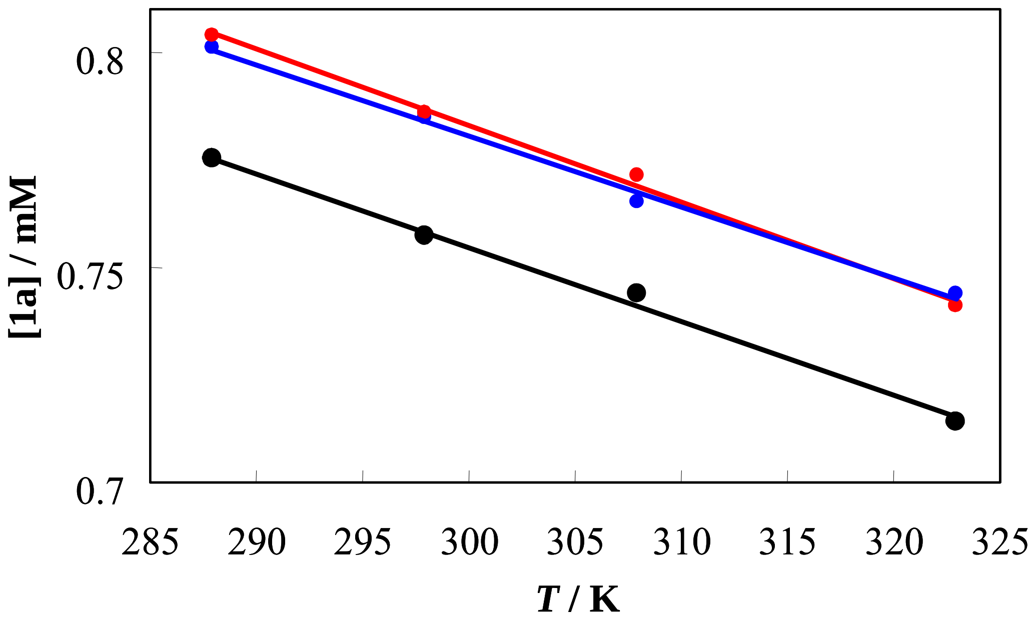

Figure S4. Effects of viscosity of solvent on decrease of **1a** by irradiation: solutions containing 1.0 mM of **1a** were irradiated using 313-nm light for 30 min, 1:1 mixtures of dimethylformamide (the viscosity coefficient is 0.34 mPa•s) – MeCN (0.92 mPa•s) [blue], N,N-dimetylacetoamide (2.14 mPa•s) – MeCN [red]. The result using a neat MeCN solution is also shown [black].

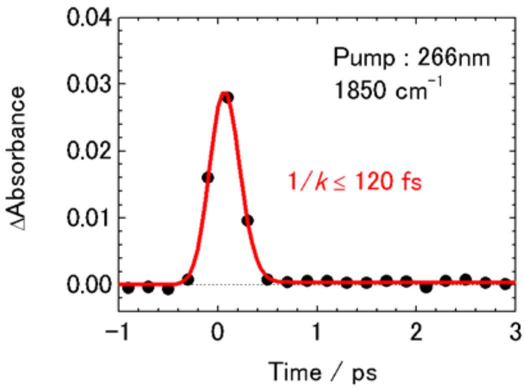

Figure S5. Time profile of the absorbance changes for **1a** in a THF solution following excitation with a 266-nm laser pulse. The monitoring wavenumber is 1850 cm^{-1}. The solid line is a curve calculated from a single exponential function convoluted with a laser function.

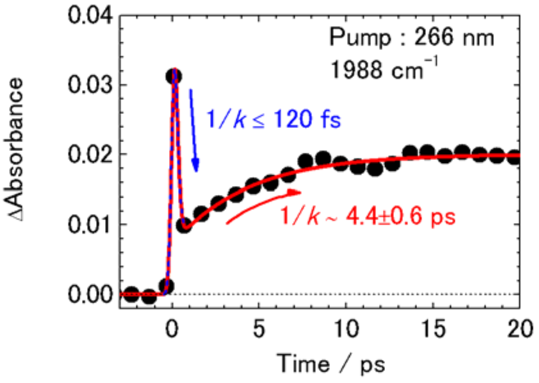

Figure S6. Time profile of the absorbance changes for **1a** in a THF solution following excitation with a 266-nm laser pulse. The monitoring wavenumber is 1988 cm^{-1}. The solid line is a curve calculated from a double exponential function convoluted with a laser function.

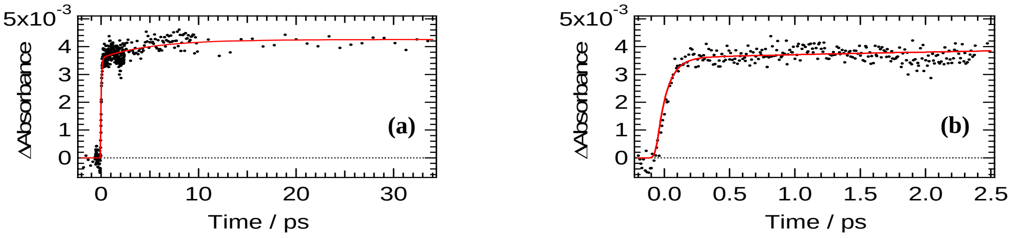

Figure S7. Time profiles of the transient absorbance of **1a** in an MeCN solution following excitation with a femtosecond 370-nm laser pulse. The monitoring wavelength was 475 nm. Solid lines are curves calculated from a triple exponential function with lifetimes of 72 ± 2 fs and 500 ± 10 fs and a 9.7 ± 0.3 ps rise.

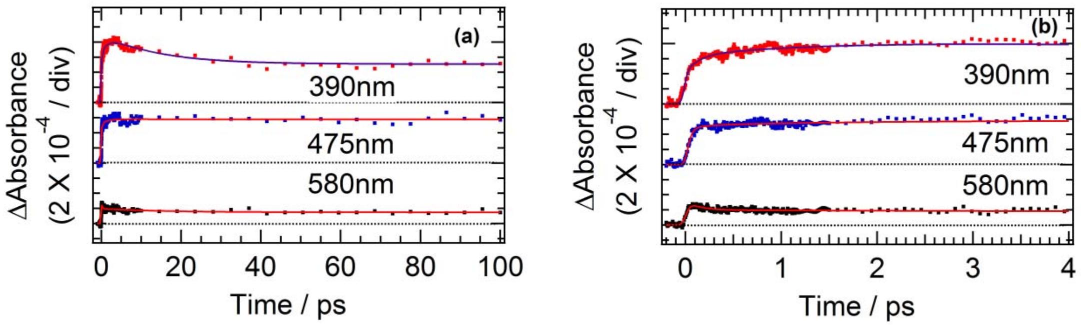

Figure S8. Time profiles of the transient absorbance of **1a** in an MeCN solution following excitation with a femtosecond 270-nm laser pulse. Monitored wavelengths were at 390, 475, and 580 nm. Solid lines are the quadruple exponential function curves with lifetimes of 30 fs, 130 fs, and 14 ps.

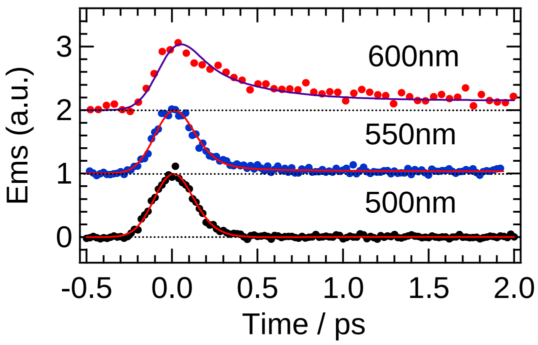

Figure S9. Time profiles of the emission up-conversion signals of **1a** in an MeCN solution following excitation with a femtosecond 400-nm laser pulse. The monitoring wavelengths are 600, 550, and 500 nm. Solid lines are results calculated from a double exponential function and residual signal with lifetimes of 71±2 fs and 360±110 fs.
